# Two distinct trajectories of clinical and neurodegeneration events in Parkinson’s disease

**DOI:** 10.1038/s41531-023-00556-3

**Published:** 2023-07-13

**Authors:** Cheng Zhou, Linbo Wang, Wei Cheng, JinChao Lv, Xiaojun Guan, Tao Guo, Jingjing Wu, Wei Zhang, Ting Gao, Xiaocao Liu, Xueqin Bai, Haoting Wu, Zhengye Cao, Luyan Gu, Jingwen Chen, Jiaqi Wen, Peiyu Huang, Xiaojun Xu, Baorong Zhang, Jianfeng Feng, Minming Zhang

**Affiliations:** 1 Department of Radiology, The Second Affiliated Hospital, Zhejiang University School of Medicine, 310000 Hangzhou, China; 2 Institute of Science and Technology for Brain-inspired Intelligence, Fudan University, 200433 Shanghai, China; 3 Key Laboratory of Computational Neuroscience and Brain-Inspired Intelligence (Fudan University), Ministry of Education, Shanghai, China; 4 MOE Frontiers Center for Brain Science, Fudan University, Shanghai, China; 5 Zhangjiang Fudan International Innovation Center, Shanghai, China; 6 Department of Computer Science, University of Warwick, Coventry, CV4 7AL United Kingdom; 7 Department of Neurology, The Second Affiliated Hospital, Zhejiang University School of Medicine, 310000 Hangzhou, China

**Keywords:** Biophysical models, Parkinson's disease

## Abstract

Increasing evidence suggests that Parkinson’s disease (PD) exhibits disparate spatial and temporal patterns of progression. Here we used a machine-learning technique—Subtype and Stage Inference (SuStaIn) — to uncover PD subtypes with distinct trajectories of clinical and neurodegeneration events. We enrolled 228 PD patients and 119 healthy controls with comprehensive assessments of olfactory, autonomic, cognitive, sleep, and emotional function. The integrity of substantia nigra (SN), locus coeruleus (LC), amygdala, hippocampus, entorhinal cortex, and basal forebrain were assessed using diffusion and neuromelanin-sensitive MRI. SuStaIn model with above clinical and neuroimaging variables as input was conducted to identify PD subtypes. An independent dataset consisting of 153 PD patients and 67 healthy controls was utilized to validate our findings. We identified two distinct PD subtypes: subtype 1 with rapid eye movement sleep behavior disorder (RBD), autonomic dysfunction, and degeneration of the SN and LC as early manifestations, and cognitive impairment and limbic degeneration as advanced manifestations, while subtype 2 with hyposmia, cognitive impairment, and limbic degeneration as early manifestations, followed later by RBD and degeneration of the LC in advanced disease. Similar subtypes were shown in the validation dataset. Moreover, we found that subtype 1 had weaker levodopa response, more GBA mutations, and poorer prognosis than subtype 2. These findings provide new insights into the underlying disease biology and might be useful for personalized treatment for patients based on their subtype.

## Introduction

Parkinson’s disease (PD) is the second most common neurodegenerative disease, and is characterized by loss of dopaminergic neurons in the substantia nigra (SN) and progressive and irreversible aggregation of misfolded α-synuclein in multiple brain regions^1^. PD varies dramatically in its manifestations, response to therapy, and long-term prognosis, suggesting the existence of PD subtypes^2^. Identification of these subtypes is crucial for better understanding the mechanisms of the disease, better prognosis, and ultimately, for delivering personalized medicine^3^.

Great efforts to identify subtypes of PD have been made, from the initial classification based on a single motor symptom, to classification based on multidomain phenotypes including motor and non-motor symptoms, neuroimaging data, and biochemical markers^3–8^. Owing to the poor reliability and difficulty in characterizing disease heterogeneity, empirical classification methods have lost favor and have been replaced by data-driven methods without a priori hypotheses^9–14^. These data-driven studies provide invaluable information toward the understanding of complex mechanisms, but have limitations. Typical data-driven subtyping methods are limited by the confound of disease stage, as the samples included in such studies are often at different timepoints in the course of the disease^12,15^. Restricted by feasibility issues such as sample size, it is difficult to stratify patients by disease duration or stage. Therefore, patients can be misclassified by data-driven subtyping methods if individuals at different timepoints or stages are included. In addition, a typical clustering analysis might not work well if the patients have different progression trajectories^16^. We speculate that this is also one of the reasons why many of these data-driven PD subtype classification systems lack reproducibility^17^. Recently, “brain-first” and “body-first” subtypes of PD have been proposed, which suggest two contrasting spatiotemporal propagation routes of α-synuclein pathology^17–19^. In the body-first subtype, the initial pathology may first appear in the enteric or peripheral autonomic nervous system. It then propagates to the medulla oblongata via the vagus nerve. Ascending pathology affects pons giving rise to Rapid Eye Movement (REM) sleep behavior disorder (RBD) before the SN is involved^19^. In the brain-first subtype, the pathology may appear in the amygdala or olfactory bulb, and then spreads to the brainstem and cortex^19,20^. In this sense, uncovering both temporal and phenotype heterogeneity when conducting a data-driven clustering analysis is somewhat meaningful.

An emerging model called Subtype and Stage Inference (SuStaIn) has been successfully used to reveal the heterogeneity and temporal complexity of neurodegenerative diseases, including Alzheimer’s disease, multiple sclerosis, and frontotemporal dementia^15,16,21–23^. The SuStaIn model is a machine-learning method that disentangles temporal and phenotypic heterogeneity to identify subtypes with distinct progression trajectories from readily accessible cross-sectional patients. Meanwhile, the model assigns each patient with the most appropriate subtype and calculates each patient’s stage within that subtype. In this model, progression trajectory refers to the sequence of abnormalities in clinical or imaging features, and stage refers to the cumulative degree of abnormalities in clinical or imaging features. If all patients of a certain type have atrophy in subcortical nuclei, but only some have atrophy in the neocortex, the SuStaIn model would infer with high confidence that subcortical atrophy occurs before neocortical atrophy in this type of patient^24^. A patient with both subcortical and neocortical atrophy would therefore have an advanced disease stage compared with those with pure subcortical atrophy.

**Fig. 1:**
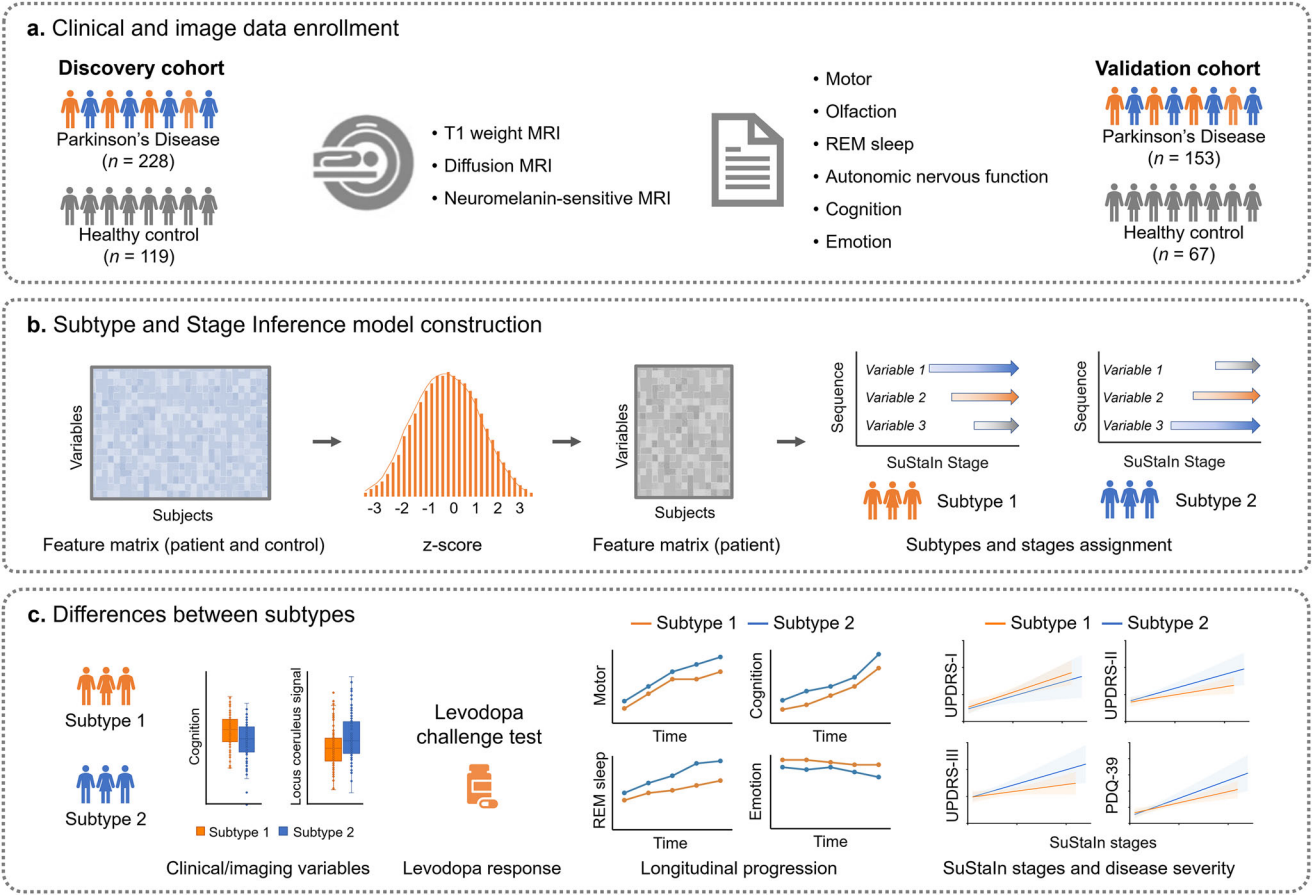
A flowchart of characterization of clinical and imaging heterogeneity in Parkinson’s disease. **a** A total of 567 individuals (347 in discovery dataset and 220 in validation dataset) were enrolled in this study; five clinical variables and 6 imaging variables were used for model construction; these imaging variables were extracted from 6 brain structures including substantia nigra, locus coeruleus, amygdala, hippocampus, entorhinal cortex, and basal forebrain by using neuromelanin-sensitive imaging and diffusion tensor imaging. **b** These variables were normalized relative to healthy control subjects using z-score; the SuStaIn model was conducted to identify distinct patterns of progression (progression trajectories of clinical and neurodegenerative events) using these variables and to cluster patients into distinct patterns (subtypes) and stages. **c** The differences of clinical/imaging variables, levodopa response, and longitudinal progression were assessed between subtypes; the consistency between the inferred SuStaIn stage and the disease severity measured by the UPDRS, PDQ-39, and other measures was evaluated. The box plot and line chart were used for visualization purposes only. Box plots with center line indicating median, bounds of boxes showing upper and lower quartile, whiskers illustrating 1.5 * interquartile range, and dots representing the distribution of raw data (minima and maxima are included). MRI magnetic resonance imaging, SuStaIn Subtype and Stage Inference, REM rapid eye movement. UPDRS Unified Parkinson’s Disease Rating Scale, PDQ-39 Parkinson’s Disease Questionnaire-39 items.

Here, we aimed to identify PD subtypes with distinct trajectories based on the above-mentioned data-driven method with multidimensional data, including comprehensive clinical evaluations and MRI features (diffusion tensor imaging and neuromelanin-sensitive MRI). These MRI measures are considered as sensitive imaging biomarkers for evaluating neurodegeneration of PD patients^6^. Furthermore, we fitted analogous multidimensional data from the Parkinson’s Progression Markers Initiative (PPMI) dataset into the SuStaIn model to test the repeatability of PD subtypes. Finally, we investigated the differences in clinical characteristics, imaging features, response to levodopa treatment, and longitudinal prognosis between these PD subtypes (Fig. 1).

## Results

Healthy controls and PD patients were not significantly different in terms of gender, age and education level. PD patients showed significant hyposmia, autonomic dysfunction, RBD symptoms, cognitive decline, and depression compared to the healthy control group. Additionally, PD patients showed significant decreased contrast-to-noise ratio (CNR) of substantia nigra (SN) and locus coeruleus (LC), and increased free-water of hippocampus and entorhinal cortex. Detailed demographics, clinical and imaging characteristics were shown in Table 1.

**Table 1:** Demographics, clinical and imaging characteristics of healthy controls and Parkinson’s disease patients in discovery dataset.

	Health control	Parkinson’s disease	*p* value
Sex (male)	119(68)	228(148)	0.157
Age (year)	61.71 ± 7.28	60.53 ± 8.56	0.20
Education (year)	9.56 ± 4.20	9.06 ± 3.85	0.268
Duration (year)	–	4.29 ± 2.82	–
LEDD	–	346.25 ± 272.41	–
UPDRS-I	–	1.54 ± 1.51	–
UPDRS-II	–	9.10 ± 5.33	–
UPDRS-III	–	22.69 ± 12.97	–
UPDRS-IV	–	0.83 ± 1.54	–
HY stage	–	2.09 ± 0.62	–
PDQ-39	–	19.76 ± 18.8	–
ADL	–	22.14 ± 5.17	–
RBDQ-HK	9.64 ± 5.77	20.75 ± 16.75	<0.001***
SCOPA-AUT	4.76 ± 3.44	9.57 ± 6.17	<0.001***
BSIT	8.50 ± 1.63	5.21 ± 2.47	<0.001***
MoCA	24.53 ± 3.36	23.11 ± 4.24	0.002**
HAMD	2.91 ± 2.99	3.80 ± 3.95	0.032*
CNR_SN_	2.50 ± 0.42	2.01 ± 0.44	<0.001***
CNR_LC_	2.26 ± 0.78	2.07 ± 0.74	0.030*
Free-water of basal forebrain	0.196 ± 0.021	0.197 ± 0.017	0.721
Free-water of entorhinal cortex	0.20 ± 0.022	0.209 ± 0.024	0.001**
Free-water of amygdala	0.172 ± 0.010	0.174 ± 0.012	0.073
Free-water of hippocampus	0.180 ± 0.016	0.185 ± 0.018	0.019*

*LEDD* levodopa equivalent daily dose, *UPDRS* Unified Parkinson’s Disease Rating Scale, *HY* Hoehn–Yahr, *PDQ-39* Parkinson’s Disease Questionnaire-39 items, *ADL* activity of daily life scale, *RBDQ-HK* Rapid Eye Movement Sleep Behavior Disorder Questionnaire (Chinese University of Hong Kong version), *SCOPA-AUT* Scales for Outcomes in Parkinson’s Disease-Autonomic, *BSIT* Brief Smell Identification Test modified for Chinese, *MoCA* Montreal Cognitive Assessment, *HAMD* Hamilton Rating Scale for Depression, *CNR*_*SN*_ contrast-to-noise ratio of substantia nigra, *CNR*_*LC*_ contrast-to-noise ratio of locus coeruleus.

*p < 0.05; **p < 0.01; ***p < 0.001.

### Clinical and neurodegeneration trajectories of subtypes

**Fig. 2:**
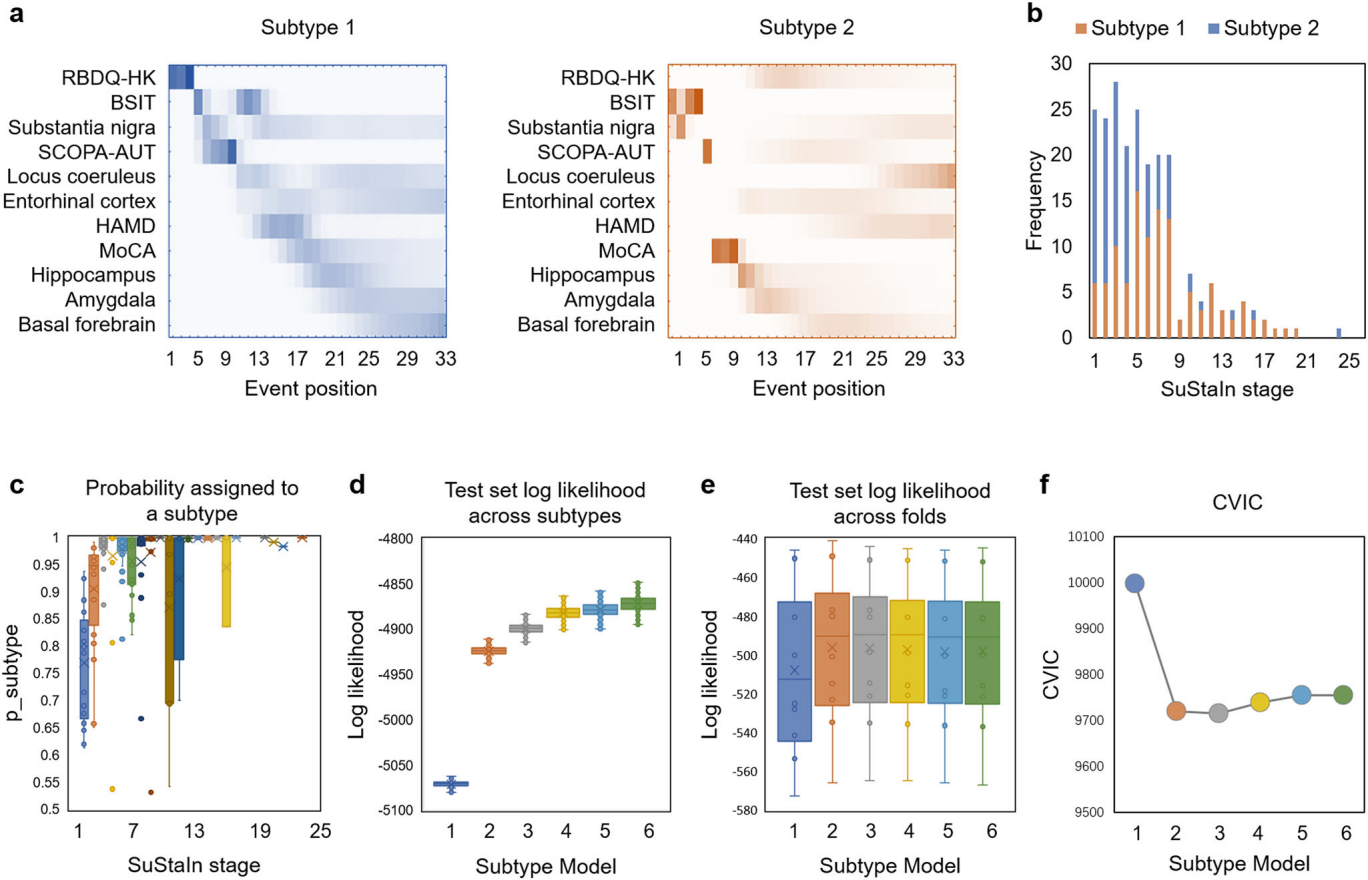
The progression patterns of Parkinson’s disease subtypes in the discovery dataset. **a** The positional density maps of two Parkinson’s disease subtypes. x axis indicates the number of events (11 variables * 3 grads). The color intensity reflects the row-wise positional density and confidence in the ordering. The location of highest color intensity means the most probable stage/sequence of this feature. **b** Distribution of two subtypes across SuStaIn stages. **c** Boxplots of probability of maximum likelihood subtype. **d** Log Likelihood across Markov chain Monte Carlo iterations. **e** Log Likelihood across 10-fold cross-validation. Log Likelihood increased dramatically from 1 subtype to 2 subtypes, but not increased slowly or even decreased from 2 subtypes to 3 or more subtypes. **f** The CVIC under different number of subtypes. Decreased CVIC illustrated improved model fit. In case of little improvement in model fit, a simpler model should be favored. Box plots with center line indicating median, bounds of boxes showing upper and lower quartile, whiskers illustrating 1.5 * interquartile range, and dots representing the distribution of raw data (minima and maxima are included). The contrast-to-noise ratio of substantia nigra and locus coeruleus, the free-water of basal forebrain, entorhinal cortex, amygdala, and hippocampus were enrolled. RBDQ-HK Rapid Eye Movement Sleep Behavior Disorder Questionnaire (Chinese University of Hong Kong version), BSIT Brief Smell Identification Test modified for Chinese, SCOPA-AUT Scales for Outcomes in Parkinson’s Disease-Autonomic, HAMD Hamilton Rating Scale for Depression, MoCA Montreal Cognitive Assessment, SuStaIn Subtype and Stage Inference, CVIC cross-validation information criterion.

Of the 228 PD patients, 114 were assigned to subtype 1 and 102 to subtype 2. Only 12 patients were categorized as stage 0, and not assigned to any subtype. Each subtype was defined by a different sequence of abnormalities in clinical symptoms and brain degeneration (Fig. 2a). Distribution of two subtypes across SuStaIn stages were shown in Fig. 2b. Boxplots of probability of maximum likelihood subtype suggested that all patients have high probability (>50%, Fig. 2c). Figure 2d, e showed the Log Likelihood across Markov Chain Monte Carlo (MCMC) iterations and 10-fold cross-validation, respectively. Figure 2f showed the cross-validation information criterion (CVIC) under different number of subtypes. A decreased CVIC illustrated an improved model fit. The CVIC showed clear decrease from one to two, but less obvious decrease from two to three subtypes. In case of very little improvement in model fit, a simpler model should be favored. Thus, the SuStaIn model with two subtypes was selected.

The SuStaIn model estimated that progression starts with classic prodromal features of PD, such as RBD, hyposmia, autonomic dysfunction, and depression, which precede cognitive impairment later in the disease course. Degeneration of the SN and LC were the earliest manifestations of the disease captured by imaging in subtype 1. These were followed by degeneration of the entorhinal cortex. Consistent with the sequence of cognitive decline, the degeneration of limbic systems such as hippocampus, amygdala, and basal forebrain occurred in the advanced stages. The first symptoms occurred in subtype 2 patients were olfaction, autonomic function, and cognition. In contrast to subtype 1, RBD and depression were confined to the latter stages of the disease. SN degeneration was the earliest sign of the disease to be imaged. Early degeneration of hippocampus and amygdala were also characteristic of this subtype. These were followed by degeneration of entorhinal cortex and basal forebrain. LC degeneration were confined to the latter stages of the disease.

Additionally, we constructed a new SuStaIn model using diffusion MRI data only (specifically measuring the free-water of substantia nigra, locus coeruleus, basal forebrain, entorhinal cortex, amygdala, and hippocampus) in the discovery dataset. The resulting progress patterns of two subtypes were consistent with above results (Supplementary Fig. 1).

### Differences of demographics, clinical variables, imaging parameters, and response to levodopa treatment between subtypes

**Fig. 3:**
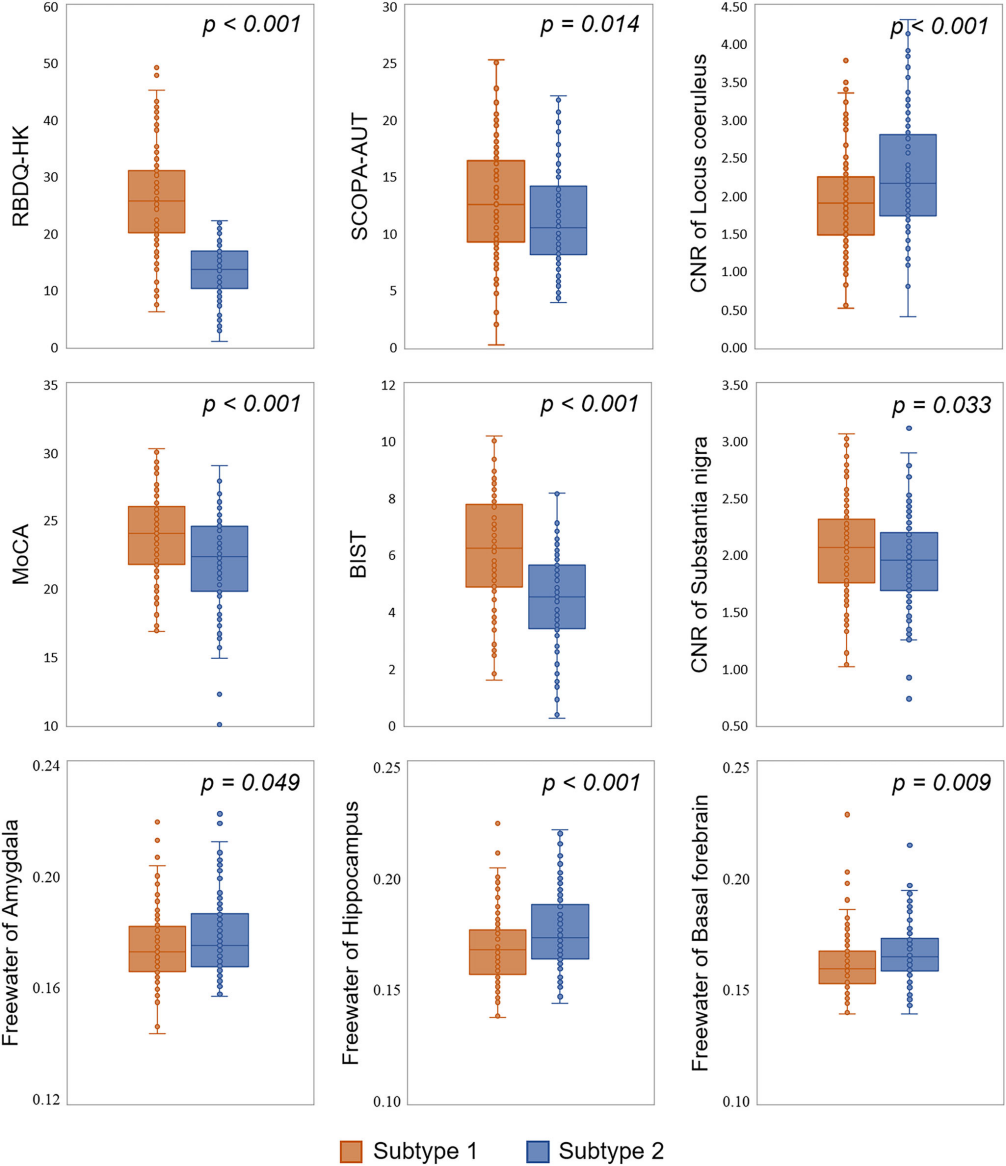
The differences of clinical and imaging features between two Parkinson’s disease subtypes in the discovery dataset. Lower CNR of locus coeruleus or substantia nigra means more severe degeneration, while higher free-water measure means more severe degeneration. Box plots with center line indicating median, bounds of boxes showing upper and lower quartile, whiskers illustrating 1.5 * interquartile range, and dots representing the distribution of raw data (minima and maxima are included). The *p* values were FDR corrected. RBDQ-HK Rapid Eye Movement Sleep Behavior Disorder Questionnaire—Chinese University of Hong Kong version, SCOPA-AUT Scales for Outcomes in Parkinson’s Disease-Autonomic, CNR contrast to noise ratio, MoCA Montreal Cognitive Assessment scale, BSIT modified Brief Smell Identification Test for Chinese. *p < 0.05; **p < 0.01; ***p < 0.001.

The two subtypes were not significantly different in their gender, age, disease duration, and levodopa equivalent daily dose (LEDD). Subtype 1 patients had lower education level (*p* = 0.005) and higher SuStaIn stage than subtype 2 (*p* < 0.001). Therefore, education and SuStaIn stage were regressed as covariates when comparing the difference of clinical variables, imaging parameters, and levodopa response between subtypes. General linear model suggested that no significant difference was found for part I, II, and III of Unified Parkinson’s Disease Rating Scale (UPDRS), and Hoehn and Yahr (HY) scores between the two subtypes. In addition, more severe RBD symptoms, autonomic dysfunction, and LC degeneration were observed in subtype 1 compared with subtype 2. By contrast, more significant declines in cognition and olfaction were found for subtype 2. More serious degeneration in SN, hippocampus, amygdala, and basal forebrain were also found for subtype 2 [Fig. 3, False discovery rate (FDR) corrected].

We compared the difference of levodopa response between the two subtypes. One hundred and twelve patients (subtype 1 = 55, subtype 2 = 57) undertook the levodopa challenge test. The two subgroups were well matched in age, gender, education level, disease duration, and LEDD. Subtype 1 patients showed significant poor levodopa responses than that of subtype 2 patients (*p* = 0.026). The difference kept significant in regression of LEDD between two subtypes (*p* = 0.025). Supplementary Table 1 shares detailed demographic information of the patients who took the levodopa challenge test.

No significant difference was observed between the two subtypes for tremor, rigidity, bradykinesia, and axial symptoms, histories of smoking, alcohol consumption, pesticide and toxic exposure, and asymmetry index of imaging variables (see Supplementary Materials for details).

### Correlations between inferred SuStaIn stage and disease severity

**Fig. 4:**
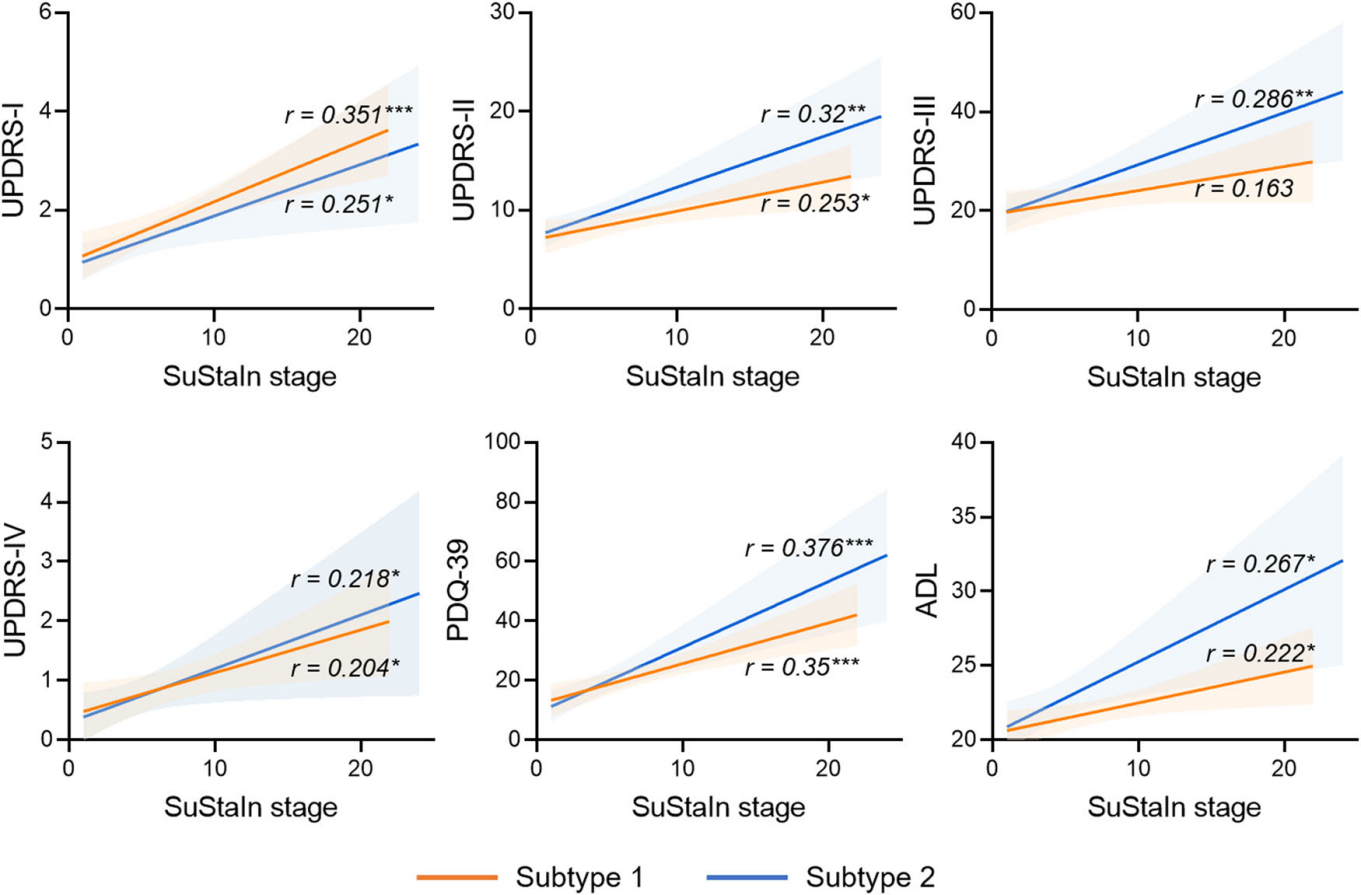
Correlations between SuStaIn stage and disease severity in two Parkinson’s disease subtypes in the discovery dataset. FDR corrected. UPDRS Unified Parkinson’s Disease Rating Scale, PDQ-39 Parkinson’s Disease Questionnaire-39 items, ADL activity of daily life scale. *p < 0.05; **p < 0.01; ***p < 0.001.

Significant correlations were found between SuStaIn stage and UPDRS-I, UPDRS-II, UPDRS-III, UPDRS-IV, Parkinson’s Disease Questionnaire-39 items (PDQ-39), and activity of daily life scale (ADL) scores in subtype 1, and UPDRS-I, UPDRS-II, UPDRS-IV, PDQ-39, and ADL scores in subtype 2 (Fig. 4, FDR corrected). These results suggest that the features used in the SuStaIn model were related to PD pathology, and that the SuStaIn stages assigned to patients were reliable.

### Subtypes in validation dataset

We validated our findings in the PPMI dataset. Healthy controls and PD patients were well matched in gender, age and education level. Detailed demographics, clinical and imaging characteristics were shown in Supplementary Table 2. One hundred and twenty-eight patients were divided into two distinct progression subtypes. The first dysfunctional signs in subtype 1 patients (*n* = 102) were in olfaction and REM sleep. These were followed by depression and autonomic dysfunction, while cognitive decline developed in the advanced stages. The initial symptoms in subtype 2 patients (*n* = 26) were cognitive decline and hyposmia. Autonomic dysfunction, RBD symptoms, and depression were limited to the latter stages of the disease. Alterations in imaging features were restricted to the end of disease stage in both subtypes. The remaining 25 patients in that dataset were categorized as stage 0. Distribution of two subtypes across SuStaIn stages, boxplots of probability of maximum likelihood subtype, MCMC iterations and 10-fold cross-validation, and the CVIC under different number of subtypes were shown in Supplementary Fig. 2. Two subtypes were determined as the optional number of subtypes.

Consistent with the discovery dataset, subtype 1 patients displayed more severe RBD (*p* = 0.044), but not survived after FDR correction, while subtype 2 patients displayed significant lower Montreal Cognitive Assessment (MoCA) score (*p* < 0.001). Three PD patients with GBA mutations and 1 PD patient with LRRK2 mutations were identified. Three patients with GBA mutations were grouped to subtype 1, and one patent with LRRK2 mutations was grouped to subtype 2. We also assessed the differences of longitudinal progression between subtypes. Linear mixed-effects model analysis suggested that subtype 1 had faster rate of progression in MDS-UPDRS-III and MDR-UPDRS-Total scores during 5 years follow up (group * time, *p* = 0.030 and *p* = 0.040, respectively, uncorrected).

No significant difference was found between the two subtypes for gender, age, education level, disease duration, MDS-UPDRS scores and motor subscales of tremor, rigidity, bradykinesia, axial symptoms, and postural instability and gait difficulty (PIGD). No significant difference was found in environmental factors, asymmetry index, dopamine transporter (DAT) binding ratios, levels of amyloid-β42, α‐synuclein, and phosphorylated tau protein in cerebrospinal fluid (Supplementary Table 4).

In addition, we found significant correlations between SuStaIn stage and MDS-UPDRS-I, MDS-UPDRS-II, and MDS-UPDRS-III scores for subtype 1 (*p* = 0.004, *p* < 0.001, and *p* = 0.015, respectively) but not for subtype 2, which might be due to the small sample size of subtype 2 (FDR corrected).

## Discussion

We identified two distinct patterns of disease progression in PD patients: subtype 1, in which RBD, hyposmia, and autonomic dysfunction are early symptoms, and subtype 2, in which hyposmia and cognitive impairment are early manifestations. Furthermore, subtype 1 was characterized by early degeneration in the SN and LC, followed by degeneration in amygdala, hippocampus, and basal forebrain as the disease progress. Subtype 2 was characterized by early degeneration in the SN, hippocampus, amygdala, and basal forebrain, with spread to the LC as the disease progressed. The validation dataset showed patterns of progression similar to the discovery dataset. Moreover, subtype 1 patients had poor levodopa response, more GBA mutations, and worse longitudinal prognosis compared with subtype 2 patients. These findings provide new insights into the heterogeneity of PD from the perspective of dynamic disease evolution, and are also potentially useful for the stratification of patients in clinical trials and practice.

According to the Braak model, PD typically begins with RBD, autonomic dysfunction symptoms, and hyposmia, which then progress to emotional and classical motor disorders before ultimately leading to cognitive impairment^24,25^. In this study, we identified a subtype of PD with an identical pattern of progress from multidimensional data by using a novel data-driven approach. Meanwhile, subtype 1 was characterized by early degeneration in SN and LC, which was followed by entorhinal cortex degeneration, and eventually spreading to the limbic system such as hippocampus, amygdala, and basal forebrain. This trajectory of brain degeneration in subtype1 is similar with the Braak staging hypothesis which describes the intracerebral formation of abnormal proteinaceous Lewy bodies and the spread of Lewy neurites from the brainstem, and midbrain to limbic cortex^25,26^. Braak also pointed that the pathology of PD may develop in the olfactory bulb and extend to adjacent regions, but these lesions do not appear to advance substantially beyond those areas^25^. Therefore, Braak concluded that although PD may have a dual-etiology (originating from the gut and olfactory bulb), it is the propagation of pathology through the brainstem that ultimately drives the disease^25^. This viewpoint is similar with the recently proposed “body-first” hypothesis^19^.

However, neuropathological and clinical evidence suggests that Braak’s hypothesis does not apply to all PD patients. We identified a subtype characterized by early hyposmia and cognitive impairment, which in the later stages is accompanied by REM sleep dysfunction. Furthermore, degeneration in the hippocampus, amygdala, and basal forebrain occurred long before it did in the LC. This subtype was consistent with the “brain-first” hypothesis^19^. A recent postmortem study further supports our findings, reporting that pathology in "brain-first" patients is initially triggered in the olfactory bulb or amygdala^20^. In addition, it has been recognized that hyposmia and cognitive impairment can occur in the prodromal stage of PD, and is included as part of the MDS research criteria for prodromal PD^27^. Our findings further suggest that early occurrence of hyposmia and cognitive impairment might not merely be a prodromal marker for increased risk of PD, instead it might herald a specific PD subtype.

The clinical and imaging profiles differed significantly between the two subtypes. Subtype 1 showed more severe RBD symptoms, autonomic dysfunction-related manifestations, and LC degeneration. By contrast, subtype 2 showed more severe cognitive impairment, hyposmia, and limbic degeneration. These characteristics of the two subtypes were well aligned with that of “body-first” and “brain-first” subtypes, respectively^18,19,28,29^, and the neuroanatomic biotypes identified using a data-driven clustering approach^14^. These authors reported two subtypes of PD, one of which was characterized by more severe RBD and autonomic dysfunction^14,18,19,28,29^. According to the “body-first and brain-first” hypothesis, α-synuclein aggregates originate in the peripheral or enteric nervous systems and gradually invade the brainstem and cortex in “body-first” subtype patients. In “brain-first” subtype patients, α-synuclein aggregation starts in the limbic system and gradually spreads to the brainstem and peripheral and enteric nervous systems. Consistent with these two subtypes, autopsies demonstrate two dominant patterns of Lewy body pathology distribution in the PD population: a brainstem-predominant pattern and a limbic-predominant pattern^29–32^. Additionally, recent study reviewed postmortem datasets and further concluded that limbic-predominant patients had initial olfactory bulb pathology and then spreads to amygdala and neighborhood regions^20^. This viewpoint could explain why hyposmia was more severe in subtype 2 patients in our current findings.

It is worth noting that previous studies also found that PD patients with RBD symptoms have worse cognitive performance^33^. Traditional clustering analysis also tends to divide patients into mild (fewer non-motor symptoms) or diffuse malignant (more severe RBD symptoms and worse cognitive performance) subtypes^10^. We considered that disease stage as a confounding factor may contribute to differences in our conclusions. For instance, a PD subtype with RBD as an early manifestation might develop cognitive impairment at an advanced stage. In contrast, a PD subtype with cognitive impairment as an early manifestation could also show the symptoms of RBD at an advanced stage. Both types of patients will reach a similar end point characterized by severe disability followed by death^7^. PD patients with RBD symptoms usually implies that the α-synuclein pathology has been present for more than 10 years, which is much earlier than the onset of motor symptoms^3^. These patients might be in the advanced pathological stages, and consequently can have more severe clinical manifestations. However, this does not imply that PD patients with RBD symptoms suffered an early cognitive decline. Accordingly, a clinical study with a large sample size suggested that subjects (but not PD patients) with RBD symptoms do not undergo a cognitive decline during a 5-year follow-up period^34^. Therefore, the clinical characteristics of the two subtypes identified in our study do not conflict with previous studies. In addition, because the subjects included in clinical studies are at different stages of the disease, most clustering algorithms will partition individuals into early-stage or advanced-stage subtypes, or mild or diffuse malignant subtypes. This is not conducive to resolving the heterogeneous patterns of progression subtypes, which are, in theory, orthogonal to disease progression itself^16^. Therefore, the SuStaIn algorithm which combining clustering with disease progression modeling was conducted to overcome this limitation.

We found that subtype 1 had a significantly poor response to levodopa replacement therapy compared to subtype 2, which suggests different pathobiological mechanisms. In line with this finding, recent animal and human studies have shown that a deficiency of the LC noradrenergic system can complicate PD symptoms and diminishes the therapeutic efficacy of levodopa^35,36^. We found that subtype 2 patients have slight severe SN degeneration when compared with subtype 1 patients, probably because “brain-first” subtype is characterized by earlier dopamine deficiency than “body-first” subtype^18^. Nonetheless, the difference is minor and necessitates a prudent explanation. The UPDRS-III scores and subscores for tremor, rigidity, bradykinesia, axial symptoms did not differ between two PD subtypes, which indicate a comparable burden of nigrostriatal dopaminergic deficit between two PD subtypes. This finding was supported by previous study, which suggested that motor symptom are not the core features for distinguishing PD subtypes^19^. Furthermore, no significant association was observed between substances such as alcohol, tobacco, and coffee and PD subtypes in our current study. And pesticide and toxic exposure may not directly affect the subtype of PD. However, to address this issue more comprehensively, further specialized epidemiological studies are required, including meticulous quantification of factors such as alcohol content, precise amount of drinking, number of cigarettes smoked, and years of smoking.

In addition to classifying patients into different subtypes, we assigned each patient for a SuStaIn stage. We found that SuStaIn stage was significantly correlated with UPDRS-I, UPDRS-II, UPDRS-III, PDQ-39, and ADL scores. Considering that these clinical variables are mostly used for evaluating disease severity from different dimensions, these results confirmed the rationality of using the SuStaIn model to assign patients into specific subtypes and stages.

The two subtypes identified from the validation dataset were consistent with the subtypes determined from the discovery dataset. Subtype 1 patients had severe RBD symptom, while subtype 2 patients had significant poorer cognitive performance. It should be noted that the results obtained from free-water measures in the validation dataset were not entirely congruent with those obtained from the discovery dataset. We speculated that the lack of additional b0 with reverse phase-encode polarity contributed to the low sensitivity in detecting the neurodegeneration of PD. It is known that susceptibility-induced distortions could significantly reduce the accuracy of signal in small brain regions^37^. The findings from validation dataset also provided new knowledge about PD subtypes. Subtype 1 patients had faster rate of progression in motor symptom and global disease severity compared to subtype 2. This was consistent with previous studies that reported a poor prognosis in PD patients with RBD symptoms^3,29^. We also found that 3 patients with GBA mutations were grouped to subtype 1, and 1 patent with LRRK2 mutations was grouped to subtype 2. This finding was consistent the proposed “body-first” and “brain-first” phenotypes^3,18^. They considered that the pathogenic mechanism introduced by LRRK2 mutations may facilitate a “brain-first” subtype, whereas the GBA mutation carriers seem to be characterized by early, marked sympathetic denervation resembling more the “body-first” phenotype. Nonetheless, we acknowledged that the sample size for this analysis was small, and further validation is needed.

No significant difference was found in DAT binding ratios between PD subtypes, which might because of the flooring effect of DAT imaging. In addition, no difference was found in levels of amyloid-β42 and pathological tau protein in cerebrospinal fluid between the two subtypes. These findings suggest that the cognitive differences may not be caused by the burden of Alzheimer’s disease-related pathology, but rather by the different pattern of progression. Taken together, these findings expand our understanding of PD heterogeneous by showing two subtypes with different temporal and phenotype progression.

We acknowledge that the current findings are not completely aligned with the pathological evidence of the "body-first and brain-first" hypothesis^31^. Measurement errors in clinical and imaging assessments, patient heterogeneity, or the different pathological susceptibility of regions of the brain, might contribute to this inconsistency. For example, objective measures, such as polysomnography and colonic transit study, are more effective in quantifying the severity of RBD and constipation compared to subjective questionnaires. Although free-water measurements may provide better pathophysiological information regarding PD, diffusion MRI scanned with single-shell (b = 1000) is not the optimal choice for estimating free-water^38^. Further studies based on multi-shell diffusion MRI scans are necessary. Future studies to investigate this inconsistency are warranted. Moreover, future studies involving peripheral system examinations such as *meta*-[^123^I]iodobenzylguanidine (^123^I-MIBG) scintigraphy and intestinal biopsies might improve the understanding of distinct progression subtypes. Incorporating a vast number of samples should increase the confidence of subtype and stage inference, and therefore, future studies with larger sample sizes will be valuable for validating the current findings. Finally, considering the complexity of multidimensional data, having a subtype and stage inference model better suited to integrating multidimensional data in the near future would be a significant development^39^.

In conclusion, we defined two PD subtypes with distinct sequences of clinical and neurodegeneration events. These subtypes exhibit differing clinical and imaging profiles and responses to treatment. These findings corroborated the classical view on disease progression reveal new insights into the non-classical trajectory of clinical symptoms and neurodegeneration. This classification might also be useful for stratifying patients entering clinical trials, and might eventually shape more individualized treatments.

## Methods

### Participants

The discovery dataset included 242 PD patients and 133 healthy control subjects. The PD patients were recruited from the Second Affiliated Hospital of Zhejiang University School of Medicine and diagnosed by experienced neurologists according to the United Kingdom Parkinson’s Disease Society Brain Bank criteria. Healthy controls were recruited from the community. Participants with missing values in clinical assessments or MRI scans that used the SuStaIn model were excluded. In addition, all images were visually inspected, and images showing intracranial mass, cerebrovascular disorders, and obvious artifacts were excluded. In total, 228 PD patients and 119 healthy controls were included.

The protocol, consent form, and other relevant documentations were approved by the ethics committee of the Second Affiliated Hospital of Zhejiang University School of Medicine before the study commenced. The study was performed in accordance with the Declaration of Helsinki and consistently with Good Clinical Practice. Before enrollment, all patients provided their written informed consent.

### Clinical and neuropsychological assessments

The UPDRS, HY, PDQ-39, and ADL scales were evaluated in PD patients. The subscores of tremors, rigidity, bradykinesia, and axial symptoms were calculated: subscore for tremor is obtained by adding the UPDRS-III items 20–21; subscore for rigidity is equal to the UPDRS-III item 22; subscore for bradykinesia is obtained by adding the UPDRS-III items 23–26 and 31; subscore for axial symptoms is obtained by adding the UPDRS-III items 27–30. All participants underwent evaluation for Brief Smell Identification Test (BSIT) modified for Chinese, Scales for Outcomes in Parkinson’s Disease-Autonomic (SCOPA-AUT) survey, Rapid Eye Movement Sleep Behavior Disorder Questionnaire—Chinese University of Hong Kong version (RBDQ-HK), MoCA test, Hamilton Rating Scale for Depression (HAMD). For PD patients, all of the above assessments were conducted during the "OFF state" (a period at least 12 h after withholding PD medications).

The self-reported lifetime histories of smoking, alcohol consumption, pesticide and toxic exposure of participants were recorded. Participants were defined as alcohol drinkers if they reported drinking at least 1 drink per week, and nondrinkers were defined as those reporting drinking 0 or <1 alcohol drink per week. Participants were defined as smokers if they reported smoking at least 1 cigarette per week, and nonsmokers were defined as those reporting smoking 0 or <1 cigarette per week. Pesticide exposure history refers to having worked in a job that required mixing, applying, or being exposed to any form of pesticide, such as herbicides, insecticides, fungicides, rodenticides, or acaricides (>6 months). Toxic exposure history was defined as exposed to some of toxic, including heavy metals, and organic pollutants (>6 months).

Additionally, 112 patients voluntarily underwent a levodopa challenge test. The UPDRS-III score was assessed during the OFF state and repeated one hour after administration of 200 mg levodopa and 50 mg benserazide (the "ON state")^40^. The levodopa response was expressed as the rate of change in the UPDRS-III score [(UPDRS-III_OFF_ − UPDRS-III_ON_)/UPDRS-III_OFF_].

### Magnetic resonance imaging data acquisition and analysis

#### Image acquisition

All participants were scanned on a GE Discovery MR750 3.0T MRI scanner. Earplugs and foam pads were used to reduce noise and head motion. High-resolution 3D T1-weighted structural MRI, diffusion tensor imaging (DTI), and neuromelanin-sensitive imaging (NM-MRI) were performed.

High-resolution 3D T1-weighted image was acquired using a fast spoiled gradient-recalled sequence: echo time (TE) = 3.036 ms; repetition time (TR) = 7.336 ms; inversion time = 450 ms; flip angle (FA) = 11°; field of view (FOV) = 260 × 260 mm^2^; matrix = 256 × 256; slice thickness = 1.2 mm; number of slices = 196 (sagittal). DTI was acquired using a spin echo-echo planar imaging sequence: TR = 8000 ms; TE = 80 ms; flip angle = 90 degrees; field of view = 256 × 256 mm^2^; matrix = 128 × 128; slice thickness = 2 mm; slice gap = 0 mm; number of slices = 67 (axial). Diffusion images were acquired from 30 gradient directions (b = 1000 s/mm2), and included five acquisitions without diffusion weighting (b = 0). An additional b = 0 acquisition with reverse phase-encode polarity was acquired for distortion correction. Neuromelanin image was acquired using a T1-weighted fast spin echo sequence: TE = 18.6 ms; TR = 600 ms; FA = 77°; FOV = 220 × 220 mm^2^; matrix = 512 × 512; slice thickness = 3 mm; slice gap = 0 mm; number of slices = 17 (axial). Scanning coverage was set from the top of basal ganglia to the bottom of the medulla oblongata. The acquisition plane was orthogonal to the brainstem.

### Image processing

#### Neuromelanin-sensitive MRI

SN and LC are vital nodes in the trajectory of PD progression. With the advent of NM-MRI, the SN and LC are visible and measurable in vivo (Supplementary Fig. 3). Therefore, we used NM-MRI to measure the integrity of SN and LC. An author (C.Z.), who was blinded to the subjects’ information, performed two manual measurements with a time interval of one month. These measurements were conducted using ITK-SNAP (https://sourceforge.net/projects/itk-snap/). The mean and standard deviation (SD) of the signal intensity (SI) in SN, LC, cerebral peduncle (CP), and pontine (PT) were calculated. The CNR_SN/LC_ was calculated as (SI_SN/LC_ − SI_CP/PT_)/SD_CP/PT_. The averaged CNR_SN/LC_ value from twice assessments were used for final analysis^41^. A lower CNR value indicates more serious degeneration. Detailed steps about manual measurements could be found in our previous study^35^.

#### Diffusion MRI

According to previous studies focusing on the trajectory of PD progression^18,20,28^, we measure the microstructural integrity of amygdala, hippocampus, entorhinal cortex, and basal forebrain. In this study, we calculated the free-water fractional volumes of them, which is an advanced diffusion MRI measure that estimates the volume fraction of extracellular space^42^. Previous studies suggested that free-water increased under the conditions of neuroinflammatory and atrophy-based neurodegeneration^43^. Free-water is considered as a more sensitive measure than traditional diffusion and volume measures in neurodegeneration^44^.

DTI data were processed using the FMRIB Software Library (FSL, http://www.fmrib.ox.ac.uk/fsl) and MRtrix3 (www.mrtrix.org). First, skulls were stripped from the DTI data for each participant. Then, topup and eddy were executed to correct susceptibility-induced distortions, eddy currents, and movements in diffusion data^45^. According to previous study, free-water was calculated by fitting a bi-tensor model^46^. The signal attenuation of the water molecules caused by and extracellular and intracellular water was predicted by this model, respectively. The derived free-water image reflects the volume fraction of extracellular space water. The steps to extract the free-water measure of above brain regions were as follows: 1) the native T1-weighted images were linearly and non-linearly aligned into Montreal Neurological Institute (MNI) standard space, and the outputs were visually inspected for errors and bad registration (4 PD patients and 1 healthy control were excluded in the following analysis); 2) the b0 images were linearly aligned to native T1-weighted images, and the free-water images were projected onto the T1-weighted images; 3) the free-water images were then linearly and non-linearly aligned into MNI space using the transformation matrix derived from T1-weighted images registration; 4) the free-water of four regions were then extracted: amygdala, hippocampus, entorhinal cortex, and basal forebrain. Amygdala, hippocampus, and entorhinal cortex atlas were acquired from the Human Brainnetome Atlas (http://atlas.brainnetome.org/). The basal forebrain (ch1-4 were combined) was defined according to the stereotaxic probabilistic maps imbedded in the Statistical Parametric Mapping (SPM, https://www.fil.ion.ucl.ac.uk/spm/) toolbox Anatomy v22c^47^. To alleviate partial volume effects, internal erosion with one voxel were implemented in all atlas.

In summary, CNR_SN_, CNR_LC_, free-water of amygdala, hippocampus, entorhinal cortex, and basal forebrain were calculated for next analysis. We also evaluated the asymmetry of imaging features included in the SuStaIn model. The formula used to calculate the asymmetry index was as follows: Asymmetry index = abs [(feature_left_ − feature_right_)/(mean (feature_left_ + feature_right_))]^48^.

### Construction of the subtype and stage inference model

#### Feature selection

Five continuous clinical features, namely RBDQ-HK, SCOPA-AUT, BSIT, MoCA, and HAMD, were included in the analysis. Motor symptoms were only assessed on PD patients, and were therefore excluded. In addition, as we aim to model the progression in established PD (rather than prodromal disease), which is not suitable for UPDRS scores (all patients have motor dysfunction) in the SuStaIn model. The inclusion of UPDRS scores in the model could result in motor dysfunction being identified as the earliest event in PD progression. At the individual patient level, this may be misleading as research conducted on populations in preclinical stages of Parkinson’s disease has revealed that changes in smell and sleep tend to appear prior to motor symptoms^24^.

MRI features included CNR_SN_, CNR_LC_, free-water of amygdala, hippocampus, entorhinal cortex, and basal forebrain, each of them was averaged over the left and right hemispheres. As a result, a final subject*feature matrix (347*11) was obtained for further analysis. The confounding variables including age, gender, and years of education were adjusted for as covariates of no interest. The adjusted features were then converted into z scores relative to the healthy controls (by first subtracting the means of healthy controls, then dividing by the standard deviation of healthy controls). As the measure of BSIT, MoCA, CNR_SN_, and CNR_LC_ decrease indicates impairment, we multiplied their z scores by −1, so that a larger z score indicates more severe impairment for all features. Finally, the normalized patient*feature matrix (228*11) was input into the model.

#### Modeling

pySuStaIn, a Python implementation of the SuStaIn algorithm^49^, was used to identify PD subtypes with distinct progression patterns and to grade a disease stage for each participant. Each feature was assigned three z scores (1, 2, and 3), which represented three continuous events (stages). In other words, each stage corresponds to a feature achieving a new z score. The advantage of this method is that it represents the continuous linear accumulation of changes caused by pathologic damage, rather than an instantaneous switch from normal to abnormal. Therefore, the pattern of progression for each disease subtype was characterized as a piecewise linear z score model, which consisted of a series of stages. Individuals were typed according to the likelihood they belonged to each SuStaIn subtype^49^, and the SuStaIn stage was determined by choosing the most probable stage^16^. The estimation of model uncertainty involved conducting 1,000,000 MCMC iterations. SuStaIn utilizes this method calculated probability for each individual into a specific stage and subtype based on their maximum likelihood^16^. Individuals with no biomarker abnormality were defined as being at stage 0 and no subtype was assigned. Finally, by iteratively increased the number of subtypes in the SuStaIn model, a less complex model (smaller number of subtypes) with lower CVIC calculated through 10-fold cross-validation was considered as the optional number of subtypes^21^. The algorithms and analytical procedures used here have been described in detail for recent studies^15,16,23^.

#### Validation dataset

We validated the reproducibility of the PD subtypes in the PPMI dataset (www.ppmi-info.org/access-data-specimens/download-data), RRID:SCR_006431^50^. For up-to-date information on the PPMI, visit www.ppmi-info.org. Subjects were scanned on Siemens 3.0 T TIM Trio scanners with the same scanning parameters. The scanning parameters of 3D T1 image were as follows: TR = 2300 ms; TE = 2.98 ms; inversion time = 900 ms; flip angle = 90°; slice number = 176; acquisition matrix = 240 × 256 and voxel size = 1 × 1 × 1 mm^3^. Diffusion tensor image was obtained with the following parameters: TR = 900 ms, TE = 88 ms, flip angle = 90°, voxel size = 2 × 2 × 2 mm^3^, slice number = 72, 64 gradient directions with a b-value of 1000 s/mm^2^. One non-gradient volume (b = 0 s/mm^2^) was also acquired. As the PPMI dataset does not contain NM-MRI data, we only calculated the free-water of SN and LC in MNI space. The SN label was obtained from the Automated anatomical labeling atlas 3^51^, and the LC label were acquired based on a LC probability atlas with peak signal coordinates observed at two SDs^52^. Consequently, the free-water of SN, LC, amygdala, hippocampus, entorhinal cortex, and basal forebrain were input into the SuStaIn model. Clinical assessments including University of Pennsylvania Smell Identification Test (UPSIT), SCOPA-AUT, RBDQ, MoCA, and Geriatric Depression Scale (GDS) were used enrolled for SuStaIn model construction. Finally, the baseline clinical and MRI data of 153 PD patients and 67 healthy controls were enrolled for validation analysis. The model construction procedures were same with the discovery dataset.

The MDS‐UPDRS and HY scale were conducted during “OFF” state. Subscore for tremor is obtained by adding the MDS-UPDRS-III items 15–18. Subscore for rigidity is equal to the MDS-UPDRS-III item 3. Subscore for bradykinesia is obtained by adding the MDS-UPDRS-III items 4–9. Subscore for axial symptoms is obtained by adding the MDS-UPDRS-III items 1 and 10–13. In addition, subscore for PIGD was calculated as the mean of 5 items, including walking, balance and freezing in MDS-UPDRS-II items 12–13, and gait, postural stability and freezing of gait in MDS-UPDRS-III items 10–12^53,54^. The DAT binding rate (caudate and putamen) and cerebrospinal fluid proteins (α‐synuclein, amyloid-β42, tau, and phosphorylated tau protein) were collected. The details of the DAT processing and cerebrospinal fluid biomarker measurements could be found in our previous study^55,56^. The gene mutation information is only available in the PPMI dataset. According to the methods used in recent studies, the LRRK2 genetic testing included G2019S and R1441G/C, N1437H mutations; GBA genetic testing included N370S (also called N409S) in all, and L483P, L444P, IVS2 + 1, and 84GG mutations; SNCA genetic testing included A53T mutation; dual mutation carriers for both LRRK2 and GBA were excluded^57–59^.

Part of the PPMI’s participants collected the information about environmental factors tied to PD risk. This is conducted through a single site, University of California, San Francisco, and PPMI participants provide consent to be followed in PPMI FOUND separately. Alcohol drinkers were defined as drunk 100 or more alcoholic drinks during lifetime; smokers were defined as smoked 100 or more cigarettes during lifetime; coffee drinkers were defined as regularly drunk caffeinated coffee, that is, at least once per week for 6 months or longer; pesticides exposure were defined as had or ever had a job in which you mixed, applied, or were exposed in some other way to any type of pesticide, including herbicides, fungicides, insecticides, rodenticides or fumigants; toxicants exposure were defined as used glues or adhesives 100 or more days at work or at home during lifetime.

The PPMI project was approved by the Institutional Review Board or Independent Ethics Committee of all participating sites in Europe, including Attikon University Hospital (Greece), Hospital Clinic de Barcelona and Hospital Universitario Donostia (Spain), Innsbruck University (Austria), Paracelsus‐Elena‐Klinic Kassel/University of Marburg (Germany), Imperial College London (UK), Pitié‐Salpêtrière Hospital (France), University of Salerno (Italy), and in the USA, including Emory University, Johns Hopkins University, University of Alabama at Birmingham, PD and Movement Disorders Center of Boca Raton, Boston University, Northwestern University, University of Cincinnati, Cleveland Clinic Foundation, Baylor College of Medicine, Institute for Neurodegenerative Disorders, Columbia University Medical Center, Beth Israel Medical Center, University of Pennsylvania, Oregon Health and Science University, University of Rochester, University of California at San Diego, and University of California, San Francisco. Informed consent was provided according to the Declaration of Helsinki.

#### Statistical analysis

Statistical Package for the Social Sciences (version 23) was used for statistical analysis. Comparisons of demographics, clinical and imaging variables between PD patients and healthy control were performed using two-sample *t*-test and chi-squared test as appropriate. General liner model was conducted to assess the difference of clinical/imaging variables, asymmetry index, levodopa response, DAT binding rate (validation dataset), and cerebrospinal fluid proteins (validation dataset) between PD subtypes. Confounding variables including gender, age, education, and SuStaIn stage were adjusted as fixed effects in linear mixed-effects model. Comparisons of environmental factors and gene mutation (validation dataset) between PD subtypes were performed using chi-squared test. Pearson correlation analysis was conducted to assess the consistency between the SuStaIn-model-inferred disease stage and UPDRS, PDQ-39, and ADL scores, which reflect the severity of the disease. FDR correction was performed for multiple comparison correction, and a two-tailed *p*-value < 0.05 was considered significant. Additionally, the linear mixed-effects model was conducted to assessed the difference of longitudinal progression between PD subtype (group*time effect, validation dataset, Supplementary Table 5).

### Supplementary information

Supplementary materials

Reporting-summary

## Data Availability

The dataset used in this study, obtained from The Second Affiliated Hospital, Zhejiang University School of Medicine, is available for qualified researchers to request for the purpose of reproducibility of the research. To access the dataset, interested researchers can contact the corresponding author directly. The PPMI dataset (https://www.ppmi-info.org/) used for this study is available for qualified researchers through application.

## Code availability

FSL (v6.0, http://www.fmrib.ox.ac.uk/fsl), MRtrix3 (www.mrtrix.org), SPM12 (https://www.fil.ion.ucl.ac.uk/spm/), and the code for SuStaIn model construction (https://github.com/ucl-pond/pySuStaIn) are open sources.
