# Author Correction: Two distinct trajectories of clinical and neurodegeneration events in Parkinson’s disease

**DOI:** 10.1038/s41531-024-00635-z

**Published:** 2024-01-16

**Authors:** Cheng Zhou, Linbo Wang, Wei Cheng, JinChao Lv, Xiaojun Guan, Tao Guo, Jingjing Wu, Wei Zhang, Ting Gao, Xiaocao Liu, Xueqin Bai, Haoting Wu, Zhengye Cao, Luyan Gu, Jingwen Chen, Jiaqi Wen, Peiyu Huang, Xiaojun Xu, Baorong Zhang, Jianfeng Feng, Minming Zhang

**Affiliations:** 1 Department of Radiology, The Second Affiliated Hospital, Zhejiang University School of Medicine, Hangzhou, 310000 China; 2 Institute of Science and Technology for Brain-inspired Intelligence, Fudan University, Shanghai, 200433 China; 3 Key Laboratory of Computational Neuroscience and Brain-Inspired Intelligence (Fudan University), Ministry of Education, Shanghai, China; 4 MOE Frontiers Center for Brain Science, Fudan University, Shanghai, China; 5 Zhangjiang Fudan International Innovation Center, Shanghai, China; 6 Department of Computer Science, University of Warwick, Coventry, CV4 7AL United Kingdom; 7 Department of Neurology, The Second Affiliated Hospital, Zhejiang University School of Medicine, 310000 Hangzhou, China

**Keywords:** Biophysical models, Parkinson's disease

Correction to: *npj Parkinson’s Disease* 10.1038/s41531-023-00556-3, published online 13 July 2023

In this article the author name Xiaojun Guan was incorrectly written as Xiaoujun Guan. The original article has been corrected.

