# Supplementary material for: Two distinct trajectories of clinical and neurodegeneration events in Parkinson’s disease
Source: NPJ Parkinsons Dis. 2023 Jul 13;9:111. doi: 10.1038/s41531-023-00556-3 (PMC10344958; doi:10.1038/s41531-023-00556-3)
Supplement: Supplementary file 1 — Supplementary materials [file 41531_2023_556_MOESM1_ESM.pdf]

Supplementary materials

Supplementary results

1. Difference of subscores for tremor, rigidity, bradykinesia, axial symptoms, and PIGD between subtypes in discovery and validation datasets.

There was no significant difference observed between the two subtypes in the discovery dataset for tremor (p = 0.070), rigidity (p = 0.244), bradykinesia (p = 0.233), and axial symptoms (p = 0.686). Similarly, in the validation dataset, no significant difference was found between the two subtypes for tremor (p = 0.931), rigidity (p = 0.753), bradykinesia (p = 0.899), axial symptoms (p = 0.47), and postural instability and gait difficulty (p = 0.544). These findings suggest that the identified PD subtypes are not primarily determined by motor subtypes.

2. Difference of environmental factors between subtypes in discovery and validation datasets.

The difference of environmental factors between PD subtypes were assessed using chi-square test. In discovery dataset, no significant difference was found in histories of smoking (p = 0.464), alcohol consumption (p = 0.068), pesticide (p = 0.480) and toxic exposure (p = 0.603).

In validation dataset, 46 PD patients were enrolled in the subgroup analysis for environmental factors. No significant difference was found in histories of smoking (p = 0.659), alcohol consumption (p = 0.659), caffeine consumption (p = 0.999),

pesticide (p = 0.999) and toxic exposure (p = 0.325) between two groups. These findings are consistent with the results from the discovery dataset.

3. Difference of GBA or LRRK2 mutation between subtypes in validation dataset.

Three PD patients with GBA mutations and 1 PD patient with LRRK2 mutations were found in the validation dataset. No patient with SNCA mutations or dual mutation for both LRRK2 and GBA was found. Three patients with GBA mutations were grouped to subtype 1 (similar to the “body-first” PD). One patent with LRRK2 mutations was grouped to subtype 2 (similar to the “brain-first” PD).

4. Difference of asymmetry between subtypes in discovery and validation datasets.

We found no asymmetry in these features for either dataset. Detailed results were listed in *Supplementary Table 3* and *4*. Previous researches about the asymmetry in brain-first versus body-first Parkinson’s Disease subtypes remains controversy, which should be further investigated.

Supplementary Tables

Supplementary Table 1. The differences in demographics and levodopa response between the two Parkinson's disease subtypes in the discovery dataset.

	**Subtype 1**	**Subtype 2**	***p* value**
Sex (male)	55 (32)	57 (36)	0.594
Age (year)	59.92±6.43	62.34±9.77	0.13
Education (year)	8.53±3.85	9.8±3.61	0.074
Duration (year)	4.97±2.99	4.17±2.9	0.15
LEDD	414.85±309.03	334.87±298.2	0.166
UPDRS-I	1.98±1.66	1.46±1.46	0.078
UPDRS-II	9.69±5.63	10.81±6.08	0.316
UPDRS-III OFF	25.05±12.37	22.28±13.97	0.269
UPDRS-III ON	16.29±10.94	12.23±8.75	0.032*
UPDRS-IV	0.96±1.37	0.67±1.73	0.317
Levodopa response	0.37±0.21	0.46±0.18	0.026*
HY stage	2.19±0.55	2.18±0.65	0.953
PDQ-39	25.46±20.07	19.98±23.13	0.191
ADL	22.04±4.65	23.18±7.55	0.343
SuStaIn stage	7.55±5.04	4.86±4.15	0.003*

LEDD: levodopa equivalent daily dose; UPDRS: Unified Parkinson's Disease Rating Scale; OFF: at least 12 hours after withholding dopaminergic medications; ON: one

hour after administration of 200 mg levodopa and 50 mg benserazide; HY: Hoehn–Yahr; PDQ-39: Parkinson's Disease Questionnaire-39 items; ADL: activity of daily life scale; SuStaIn: Subtype and Stage Inference.

Supplementary Table 2. Demographics, clinical and imaging characteristics of healthy controls and Parkinson's disease patients in the validation dataset.

	**Health control**	**Parkinson's disease**	***p* value**
Sex (male)	67 (43)	153 (97)	0.912
Age (year)	60.17±10.82	60.82±9.50	0.655
Education (year)	15.57±3.03	15.43±2.94	0.755
Duration (year)	-	1.46±1.91	-
MDS-UPDRS-I	2.48±3.42	5.17±3.75	<0.001*
MDS-UPDRS-II	0.27±0.77	5.54±4.04	<0.001*
MDS-UPDRS-III	0.59±1.31	20.71±8.99	<0.001*
HY stage	-	1.57±0.50	-
RBDQ	2.87±2.22	4.09±2.54	0.001*
SCOPA-AUT	5.88±3.71	8.97±5.93	<0.001*
UPSIT	33.55±4.06	22.43±8.45	<0.001*
MoCA	28.18±1.15	27.56±2.08	0.024*
GDS	1.54±2.80	2.33±2.53	0.039*
Free-water of substantia nigra	0.173±0.013	0.176±0.016	0.212
Free-water of locus coeruleus	0.188±0.013	0.186±0.013	0.171
Free-water of basal forebrain	0.169±0.021	0.169±0.022	0.483
Free-water of entorhinal cortex	0.193±0.020	0.190±0.019	0.254
Free-water of amygdala	0.175±0.015	0.176±0.018	0.965

Free-water of hippocampus	0.176±0.013	0.177±0.016	0.781
DAT of right caudate	2.68±0.44	1.91±0.56	<0.001*
DAT of left caudate	2.78±0.48	1.89±0.59	<0.001*
DAT of right putamen	1.97±0.40	0.82±0.34	<0.001*
DAT of left putamen	1.93±0.42	0.8±0.35	<0.001*
beta-amyloid	957.37±460.32	854.61±330.45	0.067
alpha-synuclein	1511.56±599.82	1471.07±675.82	0.675
tau	175.93±68.98	165.47±51.55	0.225
phosphorylated tau	16.07±6.43	14.36±4.73	0.039*

MDS-UPDRS: Movement Disorder Society version of Unified Parkinson’s Disease Rating Scale; HY: Hoehn–Yahr; RBDQ: Rapid Eye Movement Sleep Behavior Disorder Questionnaire; SCOPA-AUT: Scales for Outcomes in Parkinson's disease – Autonomic; UPSIT: University of Pennsylvania Smell Identification Test; MoCA: Montreal Cognitive Assessment; GDS: Geriatric Depression Scale; DAT: dopamine transporter.

Supplementary Table 3. The differences in asymmetry between the two Parkinson's disease subtypes in the discovery dataset.

**Asymmetry index**	**Subtype 1**	**Subtype 2**	***p* value**
CNR_{SN}	0.19±0.13	0.21±0.21	0.221
CNR_{LC}	0.55±0.62	0.4±0.4	0.098
Free-water of basal forebrain	0.07±0.03	0.07±0.07	0.334
Free-water of entorhinal cortex	0.1±0.07	0.11±0.11	0.277
Free-water of amygdala	0.13±0.05	0.12±0.12	0.523
Free-water of hippocampus	0.06±0.05	0.07±0.07	0.217

CNR_{SN}: contrast-to-noise ratio of substantia nigra; CNR_{LC}: contrast-to-noise ratio of locus coeruleus.

Supplementary Table 4. The differences in asymmetry between the two Parkinson's disease subtypes in the validation dataset.

Asymmetry index	Subtype 1	Subtype 2	*p* value
Free-water of substantia nigra	0.068±0.062	0.064±0.071	0.776
Free-water of locus coeruleus	0.055±0.038	0.046±0.044	0.297
Free-water of basal forebrain	0.079±0.053	0.075±0.056	0.715
Free-water of entorhinal cortex	0.073±0.063	0.092±0.07	0.191
Free-water of amygdala	0.062±0.084	0.075±0.064	0.475
Free-water of hippocampus	0.045±0.034	0.043±0.031	0.777

Supplementary Table 5. The sample size of clinical data used in the linear mixed-effects model in the validation dataset.

Follow-up visits	Follow up time (years)	Number of patients
BL	0	128
V04	1	120
V06	2	114
V08	3	116
V10	4	110
V12	5	106

Supplementary Figures

Supplementary Figure 1. The progression patterns of Parkinson’s disease subtypes obtained from to two SuStaIn models in the discovery dataset.

a: the free-water of substantia nigra, locus coeruleus, basal forebrain, entorhinal cortex, amygdala, and hippocampus were included in this model. **b:** the contrast-to-noise ratio of substantia nigra and locus coeruleus, and the free-water of basal forebrain, entorhinal cortex, amygdala, and hippocampus were included in this model. The positional density maps of two Parkinson’s disease subtypes. x axis indicates the number of events (33 events, 11 variables and 3 grads for each variable). The color intensity reflects the row-wise positional density and confidence in the ordering. The location of highest color intensity means the most probable stage/sequence of this feature. If the location with highest color intensity close to the left (early SuStaIn stage), the abnormality of this feature is considered as an early occurred event.

RBDQ: Rapid Eye Movement Sleep Behavior Disorder Questionnaire; SCOPA-AUT: Scales for Outcomes in Parkinson's disease – Autonomic; UPSIT: University of Pennsylvania Smell Identification Test; MoCA: Montreal Cognitive Assessment; GDS: Geriatric Depression Scale; SuStaIn: Subtype and Stage Inference.

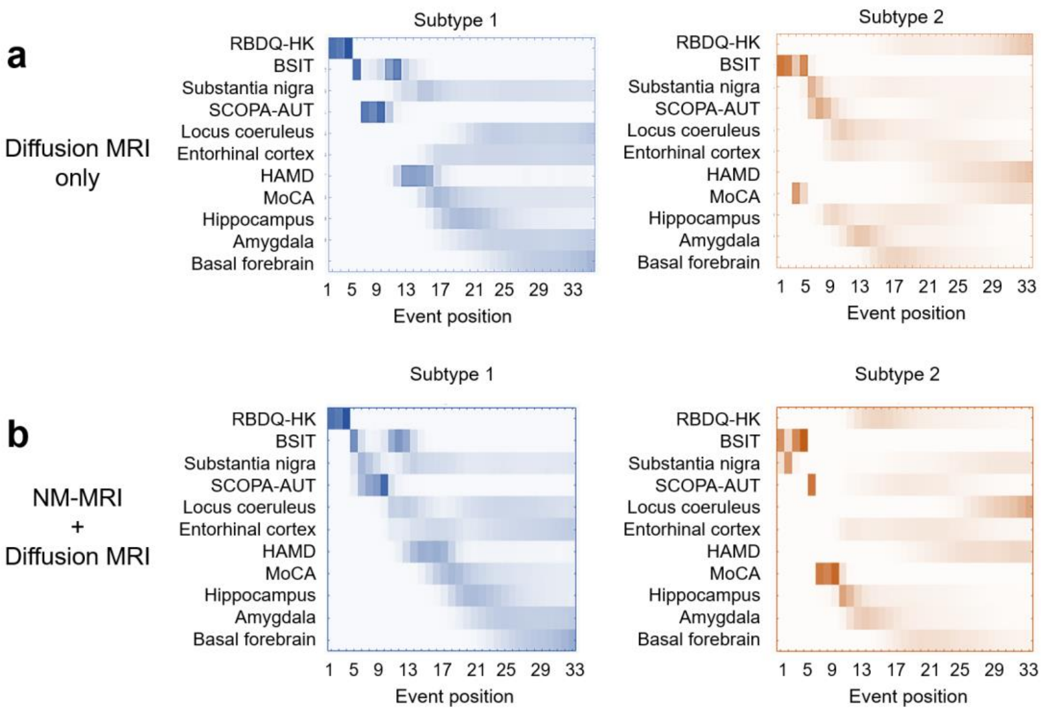

The progression patterns of two subtypes obtained from the two SuStaIn models were similar.

Supplementary Figure 2. The progression patterns of Parkinson's disease subtypes in the validation dataset.

a: the positional density maps of two Parkinson's disease subtypes. x axis indicates the number of events (33 events, 11 variables and 3 grads for each variable). The color intensity reflects the row-wise positional density and confidence in the ordering. The location of highest color intensity means the most probable stage/sequence of this feature. If the location with highest color intensity close to the left (early SuStaIn stage), the abnormality of this feature is considered as an early occurred event. **b:** distribution of two subtypes across SuStaIn stages. **c:** boxplots of probability of maximum likelihood subtype. **d:** Log Likelihood across Markov chain Monte Carlo iterations. **e:** Log Likelihood across 10-fold cross-validation. Log Likelihood increased dramatically from 1 subtype to 2 subtypes, but not increased slowly or even decreased from 2 subtypes to 3 or more subtypes. **f:** the CVIC under different number of subtypes. Decreased CVIC illustrated improved model fit. The CVIC showed clear decrease from one to two, but less obvious decrease from two to three subtypes. In case of little improvement in model fit, a simpler model should be favored. Thus, the SuStaIn model with two subtypes was selected. Box plots with centre line indicating median, bounds of boxes showing upper and lower quartile, whiskers illustrating 1.5 * interquartile range, and dots representing the distribution of raw data (minima and maxima are included).

The free-water of substantia nigra, locus coeruleus, basal forebrain, entorhinal cortex,

amygdala, and hippocampus were included in the model. RBDQ: Rapid Eye Movement Sleep Behavior Disorder Questionnaire; SCOPA-AUT: Scales for Outcomes in Parkinson's disease – Autonomic; UPSIT: University of Pennsylvania Smell Identification Test; MoCA: Montreal Cognitive Assessment; GDS: Geriatric Depression Scale; SuStaIn: Subtype and Stage Inference; CVIC: cross-validation information criterion.

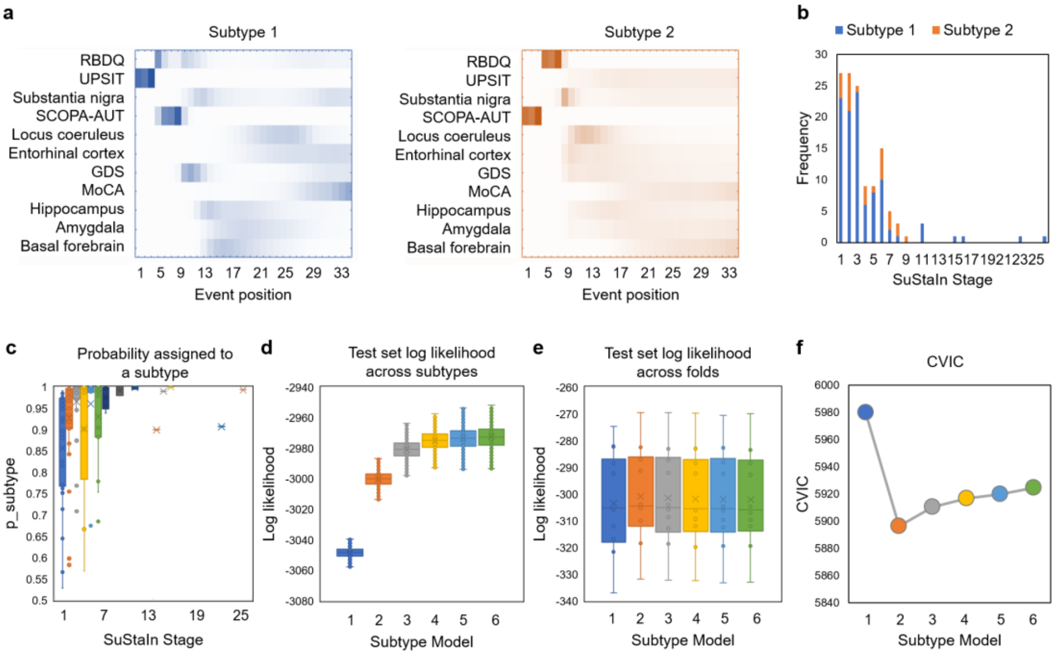

Supplementary Figure 3. The location of substantia nigra and locus coeruleus. The substantia nigra and locus coeruleus of one participant were shown in three consecutive slices. Inside the red circles are substantia nigra and locus coeruleus. Inside the white circles are cerebral peduncles and pontine (reference regions).

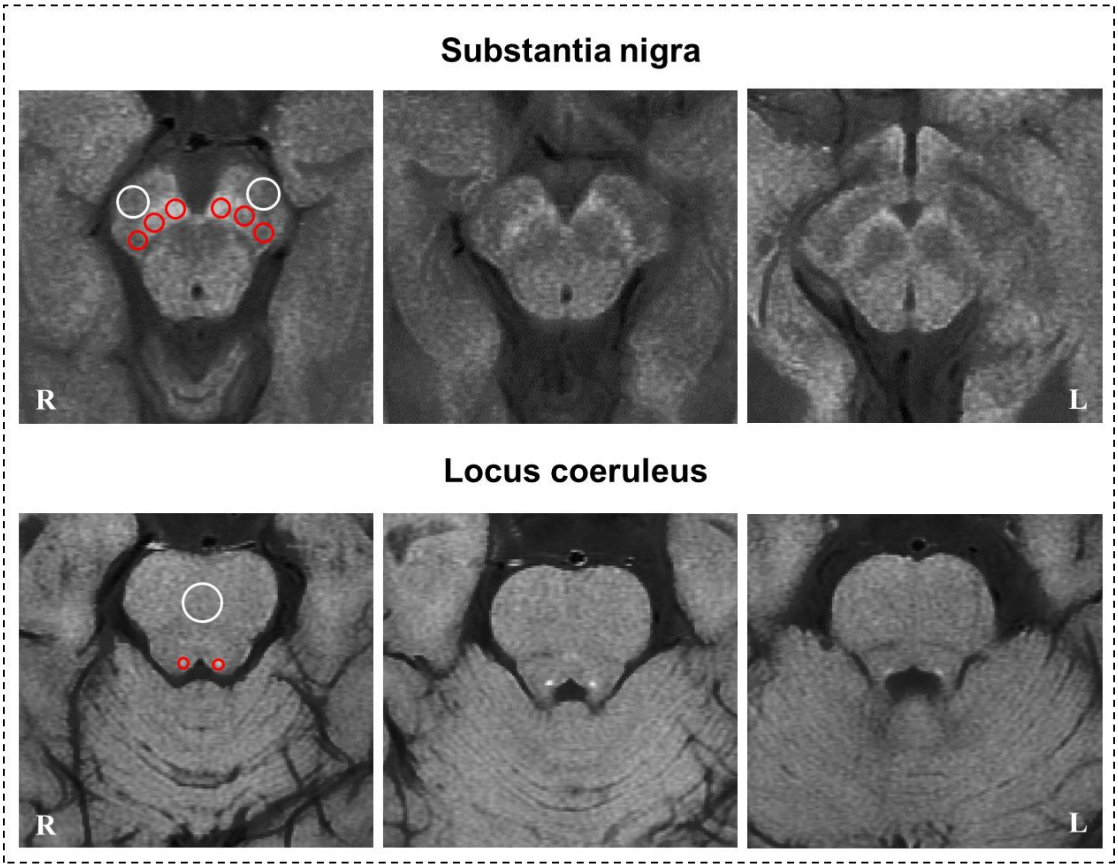

R: Right; L: Left.
